# Assessing the Impact of Comorbid Hypercalcemia on Inpatient Outcomes of Patients With Diffuse Large B-cell Lymphoma During Admission for Chemotherapy

**DOI:** 10.7759/cureus.54769

**Published:** 2024-02-23

**Authors:** Dennis D Kumi, Vaishali Deenadayalan, Samuel M Odoi, Badri Aryal, Ekrem Turk, Ayobami Olafimihan, Khaldun Obeidat, Jay Vakil, Navika Chhabra, Maryam Zia

**Affiliations:** 1 Internal Medicine, John H. Stroger, Jr. Hospital of Cook County, Chicago, USA; 2 Medicine, Kreiskrankenhaus Bergstrasse GmbH, Heppenheim, DEU; 3 Internal Medicine, John H. Stroger, Jr Hospital of Cook County, Chicago, USA; 4 Hematology/Oncology, John H. Stroger, Jr. Hospital of Cook County, Chicago, USA

**Keywords:** tumor lysis syndrome, adverse inpatient outcomes, chemotherapy admission, diffuse large b-cell lymphoma, hypercalcemia

## Abstract

Introduction

Diffuse large B-cell lymphoma (DLBCL) may be complicated by hypercalcemia at various stages of treatment. The impact of hypercalcemia on chemotherapy admission outcomes in DLBCL is not well described.

Methods

In a retrospective analysis, using the National Inpatient Sample database (2018 - 2020), patients with DLBCL admitted for chemotherapy were dichotomized based on the presence of hypercalcemia. Our primary outcome was all-cause mortality. Secondary outcomes included length of stay (LOS), total charge, rate of acute kidney injury (AKI), tumor lysis syndrome (TLS), hyperkalemia, metabolic acidosis, acute encephalopathy, septic shock, Clostridiodes difficile infection, acute respiratory failure, and venous thromboembolic events (VTE).

Results

We identified 78,955 patients, among whom 1,375 (1.74%) had hypercalcemia. Hypercalcemia was associated with higher odds of all-cause mortality (aOR:3.05, p-value:0.020), TLS (aOR:8.81, p-value<0.001), acute metabolic encephalopathy (aOR:4.89, p-value<0.001), AKI (aOR:5.29, p-value<0.001), hyperkalemia (aOR:2.84, p-value:0.002), metabolic acidosis (aOR:3.94, p-value<0.001) and respiratory failure (aOR:2.29, p-value:0.007) and increased LOS by 1 day and total charge by 12, 501 USD.

Conclusions

In patients with DLBCL admitted for inpatient chemotherapy, those with hypercalcemia compared to a cohort without had higher odds of; all-cause mortality, TLS, AKI, acute encephalopathy, acute metabolic acidosis, hyperkalemia, and acute respiratory failure as well as higher LOS and total charge.

## Introduction

Aggressive non-Hodgkin's lymphomas (NHL) account for three to five percent of all B-cell non-Hodgkin's lymphomas and are associated with high-risk clinical features, including central nervous system involvement. Adverse outcomes are higher for tumors with high-risk mutations, such as in patients with the so-called “double hit” lymphomas which carry both myc and bcl2 translocations [1]. Diffuse large B-cell lymphoma (DLBCL) is a form of aggressive NHL that makes up 31% of cases in Western nations [2]. Around 40% of patients with DLBCL have extranodal disease and up to a third of patients have B symptoms at presentation [3]. Several prognostic tools have been designed to predict mortality in patients with DLBCL such as the International Prognostic Index (IPI) [4]. 

Malignancy-associated hypercalcemia has been described as an ominous feature of both solid and hematological malignancies. It is usually related to an over-expression of parathyroid hormone (PTH)-related peptide and extra-renal 1,25 dihydroxy-vitamin D. Other humoral mediators have been described including local dysregulation in RANK ligand and osteoprotegerin as well as over-expression of macrophage inflammatory protein-1 alpha (MIP1α) and tumor necrosis factor (TNF) [5-8]. Studies from solid tumors have linked hypercalcemia of malignancy with a 1-year survival of less than 25% and studies with multiple myeloma have also equated hypercalcemia as a marker of high-risk tumor cytogenetics [9,10]. In lymphoproliferative disease, most humoral hypercalcemia is related to excessive 1,25 dihydroxy-vitamin D. Hypercalcemia complicates up to 7.4% of non-Hodgkin lymphoma [11]. Studies have shown that the presence of hypercalcemia at the time of diagnosis is associated with poorer prognosis and patients with DLBCL who present with hypercalcemia often have advanced disease, extensive extranodal involvement, more B symptoms, higher levels of LDH and β2‐microglobulin as well as lower levels of albumin and hemoglobin [12,13]. Additionally, hypercalcemia has been found to be related to disease relapse and death and is also related to lower progression-free survival (PFS) and overall survival (OS) [14]. 

There is a paucity of data on inpatient outcomes among patients with DBCL undergoing chemotherapy and the impact of comorbid hypercalcemia on these outcomes. We hypothesized that beyond its role as a predictor of the aggressiveness of tumor biology, hypercalcemia portends increased risk in many ways during chemotherapy admission. These may relate to the metabolic and cytotoxic injury of excessive calcium phosphate deposition as well as the impact of treatment for hypercalcemia itself.

## Materials and methods

Study design and data source

A retrospective analysis was performed using data from the combined National Inpatient Sample (NIS) from 2018 to 2020. Using appropriate ICD-10 codes, adults (age ≥ 18 years of age) with diffuse large B-cell lymphoma (DLBCL) hospitalized for chemotherapy were identified and dichotomized into two cohorts based on the presence of a secondary diagnosis of hypercalcemia. The NIS is a large administrative database provided by the Agency for Healthcare Research and Quality (AHRQ), which contains data from approximately a 20% sample of inpatient hospitalizations in the United States. The NIS includes information on the principal diagnosis, which is the main reason for hospitalization identified by the primary International Classification of Diseases, Tenth Revision (ICD-10) code, as well as secondary diagnoses recorded during the hospitalization. This database provides a valuable resource for researchers to examine patterns of care and outcomes in a nationally representative sample of hospitalized patients in the U.S. [15]. 

Patient population and study outcomes

The study population consisted of all inpatient hospitalizations recorded in the NIS from 2018 to 2020. Out of this, we sampled patients with a principal diagnosis of diffuse large B-cell lymphoma (DLBCL) who were admitted for chemotherapy. We analyzed patient characteristics such as demographics, hospital-level characteristics, and relevant medical comorbidities. The primary outcome was all-cause inpatient mortality. Secondary outcomes of our study were the length of stay (LOS), total charge, rate of acute kidney injury (AKI), tumor lysis syndrome (TLS), hyperkalemia, metabolic acidosis, acute encephalopathy, septic shock, Clostridiodes difficile infection, acute respiratory failure, and acute venous thromboembolic events. In a secondary analysis, we assessed a linear relationship between LOS up to the first two weeks (14 days) and two composite outcomes. Firstly, with a composite of TLS-related complications (TLS, AKI, hyperkalemia, and metabolic acidosis) and subsequently between LOS and respiratory failure-related complications (respiratory failure, acute pulmonary edema, mechanical ventilation) between both cohorts. Relevant ICD-10 codes used are listed in the Appendices Table 3. 

Statistical analysis

We performed statistical analyses using Stata, version 17 (Stata Corp, College Station, USA) standard edition. A univariate logistic regression analysis was conducted using all available variables and comorbidities to calculate unadjusted odds ratios for the primary outcome. We included all variables with P-values less than 0.1 in a multivariate logistic regression model to control for confounders. In this study, relevant confounders adjusted for were age, race, hospital location and teaching status, Charlson comorbidity index as well as specific comorbidities including CKD, history of stroke, HIV, and protein energy malnutrition. To estimate the effect sizes, we used odds ratios and mean differences. The Whitney Mann-U test for non-parametric data was used to compare group differences in continuous variables while group differences in categorical variables were compared using Pearson’s chi-square analysis and Fisher exact test. All tests were double-sided. We considered outcomes with P-values less than 0.05 and a 95% confidence interval to be statistically significant. To adjust for the comorbidity burden, we used the Charlson Comorbidity Index. 

Ethical considerations

All patient data in the NIS are de-identified and the data is publicly available. Therefore, we did not seek institutional review board approval for this study.

## Results

Baseline characteristics

Total chemotherapy admissions for patients with diffuse large B-cell lymphoma was 78,955. Among these 1,375 had hypercalcemia, representing 1.74%. Of the patients with DLBCL who had hypercalcemia, 0.5% had hyperparathyroidism. Among patients with DLBCL who were admitted for chemotherapy, patients with hypercalcemia had an average age of 66 years, were significantly older by an average of six years (p-value<0.001), and included a higher prevalence of white race (80.67% vs 70.55%) but fewer Hispanic race (5.58% vs 11.5%) compared to those without hypercalcemia. There was a higher prevalence of protein-energy malnutrition (17.45% vs 8.33%), coronary artery disease (16.36% vs 8.49%), and chronic kidney disease (18.55% vs 6.34%) among patients with hypercalcemia. There was no difference in the prevalence of HIV infection between those with and without hypercalcemia (7.27% vs 9.42%, p-value:0.245). There was no difference in terms of hospital-level characteristics such as geographic location (urban vs rural) as well as academic vs community status between those with hypercalcemia and those without. Details of baseline characteristics and univariate analysis of mortality are presented in Table 1 below. 

**Table 1:** Baseline patient demography, comorbidity, hospital characteristics and univariate analysis for all-cause mortality among patients with DLBCL admitted for chemotherapy with or without hypercalcemia. DLBCL: diffuse large B-cell lymphoma; CKD: chronic kidney disease; PEM: protein-energy malnutrition; COPD: chronic obstructive pulmonary disease; HIV: human immunodeficiency virus ** Statistically significant p-value <0.05

Baseline characteristics	DLBCL without hypercalcemia n = 77,580	DLBCL with hypercalcemia n = 1,375	P-Value **	
				Demography and hospital characteristics				
Age/years	60	66	<0.001	
Sex (Male)	59.06%	59.27%	0.956	
Race			0.027	
White	70.55%	80.67%		
Black	8.14%	5.95%		
Hispanic	11.5%	5.58%		
Asian/pacific islander	5.52%	4.09%		
Native American	0.39%	0.37%		
Other	3.90%	3.35%		
Charlson comorbidity index			0.045	
0-2	54.56%	42.55%		
3 or more	45.44%	57.45%		
Median Household income (quartile)			0.533	
1st quartile	20.38%	16.97%		
2^nd^ quartile	23.64%	25.83%		
3^rd^ quartile	26.10%	26.20%		
4^th^ quartile	29.88%	31.00%		
Expected primary payer			<0.001	
Medicare	41.46%	59.93%		
Medicaid	12.11%	5.88%		
Private insurance	43.82%	32.72%		
Self-pay and others	2.61%	1.47%		
Hospital region			0.024	
Northeast	22.67%	18.91%		
Midwest or North central	23.87%	23.64%		
South	30.29%	26.18%		
West	23.17%	31.27%		
Hospital bed size			0.181	
Small	9.87%	10.18%		
Medium	17.65%	22.18%		
Large	72.49%	67.64%		
Hospital location and teaching status			0.193	
Rural	1.13%	1.82%		
Urban non-teaching	5.68%	8.00%		
Urban teaching	93.19%	90.18%		
Patient comorbidities				
Hypertension	37.99%	40.73%	0.402	
Coronary artery disease	8.49%	16.36%	<0.001	
History of stroke	0.37%	0.36%	0.992	
Diabetes Mellitus	18.69%	23.27%	0.059	
CKD	6.34%	18.55%	<0.001	
Cirrhosis	0.59%	1.45%	0.066	
Hypothyroidism	10.18%	12.00%	0.387	
Obesity	8.29%	8.00%	0.858	
PEM	8.33%	17.45%	<0.001	
COPD	3.66%	4.73%	0.386	
HIV infection	9.42%	7.27%	0.245	

Primary and secondary outcomes

Results from our multivariate analysis of adverse outcomes based on the presence of comorbid hypercalcemia among patients with DLBCL admitted for chemotherapy after adjusting for potential confounders including age, race, hospital location, and teaching status, Charlson comorbidity index, CKD, history of stroke, HIV, and protein energy malnutrition are summarized in Table 2.

**Table 2:** Results of multivariate analysis of adverse clinical outcomes among patients with DLBCL admitted for chemotherapy with or without hypercalcemia. DLBCL: Diffuse large B-cell lymphoma; VTE: venous thromboembolic events. ◊◊ Adjusted for age, race, hospital location and teaching status, Charlson comorbidity index, specific comorbidities including CKD, history of stroke, HIV, and protein energy malnutrition. ** Statistically significant p-value <0.05

OUTCOMES	DLBCL with hypercalcemia (n = 1,375)	DLBCL without hypercalcemia (n = 77,580)	Adjusted Odds ratio. ◊◊ Adjusted difference◊◊ (95% Confidence interval)	P-value **
All-cause mortality	1.82% (n=25)	0.45% (n=349)	3.05 (1.20 – 7.78)	0.020
Acute Kidney Injury	33.09% (n=455)	7.62% (n=5,912)	5.29 (3.98 – 7.03)	<0.001
Hyperkalemia	3.64% (n=50)	1.11% (n=861)	2.84 (1.47 – 5.47)	0.002
Acute metabolic acidosis	6.18% (n=85)	1.52% (n=1,179)	3.94 (2.37 – 6.56)	<0.001
Metabolic encephalopathy	3.27% (n=45)	0.48% (n=372)	4.89 (2.36 – 10.15)	<0.001
Tumor Lysis syndrome	11.27% (n=155)	1.27% (n=985)	8.81 (5.68 – 13.65)	<0.001
Respiratory failure	4.36% (n=60)	1.49% (n=1,156)	2.29 (1.26 – 4.17)	0.007
Acute VTE	4.73% (n=65)	4.89% (n=3,794)	1.00 (0.57 – 1.75)	0.996
Clostridioides difficile colitis	0.36% (n=5)	0.82% (n=636)	0.40 (0.06 – 2.85)	0.359
Septic shock	1.09% (n=15)	0.41% (n=318)	2.31 (0.72 – 7.40)	0.159
Length of stay	6 days	5 days	1.00 day (0.4 – 1.64)	0.002
Total Charge	US$ 106,855.	US$ 93,090	US$ 12,501 (8541-33,544)	0.004

**Figure 1:**
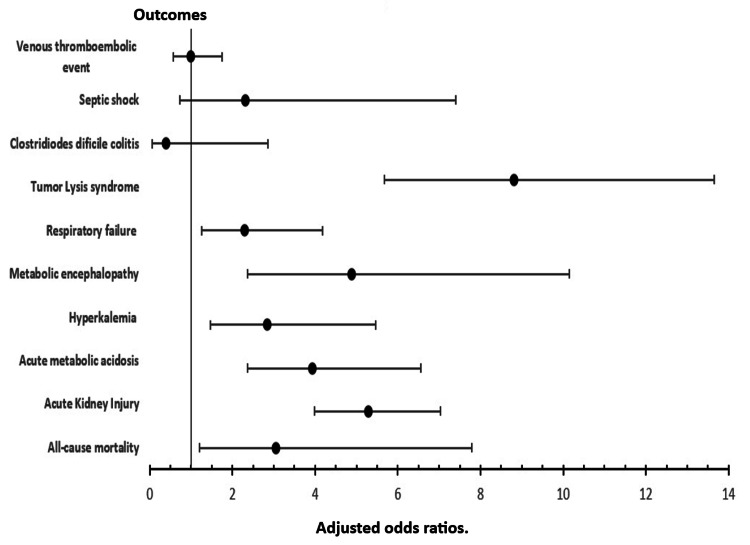
A forest Plot of odds ratios for adverse clinical outcomes during admissions for chemotherapy among aatients with DLBCL and hypercalcemia. DLBCL: Diffuse Large B-Cell Lymphoma Adjusted for age, race, hospital location and teaching status, Charlson comorbidity index, specific comorbidities including CKD, history of stroke, HIV, and protein energy malnutrition.

Among DLBCL patients hospitalized for chemotherapy, comorbid hypercalcemia was associated with 3-fold higher odds of all-cause mortality (aOR: 3.05, p-value:0.020) compared to other cohorts without hypercalcemia. There were increased odds on several other clinical outcomes studied including an 8-fold increase in tumor lysis syndrome (aOR: 8.81, p-value<0.001), a 5-fold increase in acute metabolic encephalopathy (aOR:4.89, p-value<0.001), 5-fold increased odds of AKI (aOR:5.29, p-value<0.001), 3-fold increased odds of hyperkalemia (aOR:2.84, p-value:0.002), a 4-fold increase in metabolic acidosis (aOR:3.94, p-value<0.001) and a 2-fold increase in respiratory failure (aOR:2.29, p-value:0.007). Additionally, patients with hypercalcemia had an increased length of stay by one day and an increase in total charge by 12, 501 USD compared to the cohort without hypercalcemia. Some clinical outcomes were however not different between groups irrespective of the presence of hypercalcemia, including the rate of acute VTE, septic shock, and Clostridiodes difficile infections. Figure 1 gives a graphical presentation of effect size in odds ratios of various adverse outcomes based on the presence or absence of hypercalcemia and their statistical significance. 

**Figure 2:**
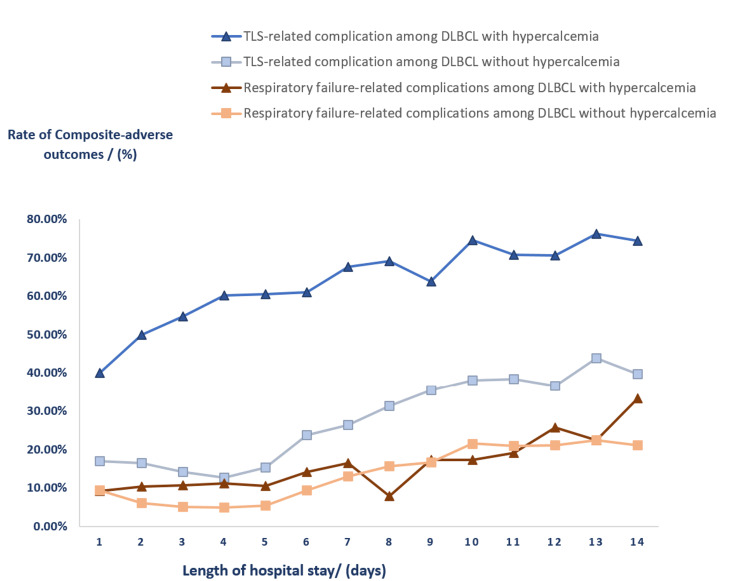
A Plot of Rate of Composite Outcomes of TLS-related Complications and Respiratory Failure-related Complications by Length of Stay Among Patients with DLBCL During Chemotherapy Admission. DLBCL: Diffuse large B-cell lymphoma; TLS: Tumor Lysis syndrome; TLS-related complications: composite of tumor lysis syndrome, acute kidney injury, hyperkalemia, and metabolic acidosis; Respiratory-failure related complication: composite of acute respiratory failure, acute pulmonary edema, and mechanical ventilation.

From our secondary analysis as graphically depicted in Figure 2, we observed that there was a linear positive correlation between LOS and frequency of composite TLS-related complications as well as composite complications related to respiratory failure. At various levels of LOS, frequencies of the two composite adverse outcomes remained higher among patients with hypercalcemia compared to those without.

## Discussion

There exists extensive knowledge about the overall impact of hypercalcemia as a prognostic factor on the aggressiveness of NHL and DLBCL. Large real-world data on the impact of hypercalcemia on inpatient outcomes of patients admitted for chemotherapy is limited. The NIS is an ideal database for studying acute inpatient adverse outcomes in such patients.

Baseline characteristics

From our study, we reported a prevalence of hypercalcemia among patients with DLBCL admitted for chemotherapy to be 1.74% over the study period. This mimicked the overall prevalence among patients with non-Hodgkin’s lymphoma but was lower than the prevalence of DLBCL reported by other literature. For instance, Abadi and colleagues estimated that among patients with newly diagnosed de-novo DLBCL, the prevalence of hypercalcemia stood at 18% [12]. Similar analyses by Gauchy et al, investigating de novo DLBCL and transformed lymphoma noted that at the time of diagnosis, 23% had hypercalcemia [13]. These studies estimated the prevalence of hypercalcemia in smaller samples of 250 and 305 patients respectively at the time of initial diagnosis. Our estimates are on all chemotherapy admissions of a much larger and heterogeneous population across the study period. These most likely include many patients who may have had some prior treatment for the initial aggressive stage of the disease. Data on larger inpatient samples is lacking. We are however cognizant of potential coding errors associated with large administrative databases and the risk of misclassification bias. We observed that patients with hypercalcemia were older than their counterparts during admission for chemotherapy. Maartense et al had shown that older age was associated with worse outcomes in DLBCL even after adjusting for IPI score. [16]. These differences in baseline characteristics were therefore accounted for in our multivariate analysis of primary and secondary outcomes.

Primary and secondary outcomes: Our study reported threefold higher odds of all-cause mortality (aOR: 3.05, p-value:0.020) compared to the other cohorts without hypercalcemia. Earlier studies had established a link between hypercalcemia and disease progression and death [14]. A similar non-favorable association between hypercalcemia and lower survival among patients with DLBCL has been observed in other studies [12,13]. Our findings of higher odds of mortality is likely more related to non-PTH dependent hypercalcemia of malignancy due to a very low prevalence of hyperparathyroidism in our study population. This finding is in keeping with reported negative prognostic values of malignancy-related hypercalcemia [17,18]. Hypercalcemia of malignancy is among the most common paraneoplastic syndromes and has often served as a prognostic indicator in several cancers. From a similar analysis by Abadi et al, the overall survival and progression-free survival among patients with DLBCL were lower in those with hypercalcemia [12]. Several reasons may underpin lower survival. Firstly, hypercalcemia is known to be associated with a large tumor burden or increased aggressiveness of the oncogenic mutations [19]. Patients with hypercalcemia often were older and had higher comorbidity. A study by Deenadayalan et al had found that protein energy malnutrition (PEM) was independently associated with higher inpatient mortality among patients with DLBCL admitted for chemotherapy [20]. Despite accounting for comorbidity burden and age, our study found increased odds of mortality among those with hypercalcemia. We observed that patients with hypercalcemia had several inpatient complications related to their malignancy and its treatment that could likely increase mortality. We used a large, population-weighted database and accounted for potential confounders thus limiting the risk of selection bias. We however remain at risk of misclassification bias as with most large administrative databases that are reliant on ICD-coding, and these are factored into our conclusions. 

From our study, critical complications including TLS, AKI, metabolic acidosis, and hyperkalemia were higher among patients with hypercalcemia. These are likely related to side effects of more aggressive chemotherapy and the impact of a large tumor burden. The risk of TLS in DBCL has also been related to advanced age at presentation and tumor bulk. [21]. Hypercalcemia may independently cause nephrocalcinosis which may further worsen electrolyte and acid-base complications [22,23]. Our observation of higher odds of acute encephalopathy reflects the impact of several factors including the effect of primary lymphoma, central nervous system (CNS)involvement, CNS paraneoplastic syndrome, the effect of hypercalcemia and metabolic encephalopathy due to electrolyte derangement, acid-base disorders, and uremia, as well as hypercalcemia related posterior reversible encephalopathy syndrome. [24, 25] There is also the concern of side effects of treatment of hypercalcemia with hyperhydration such as volume overload and acute respiratory failure [26]. From our secondary analysis as depicted in Figure 2, we observed that inpatient complications increased in frequency with increased LOS. Though this may simply reflect disease severity, it may also support the assertion that inpatient interventions including treatment of hypercalcemia may have led to the development of newer complications.

Finally, we observed a significantly higher total charge and length of stay among patients with hypercalcemia. Data related to health care utilization and the cost of treatment of DLBCL are limited. It is reported that patients with aggressive lymphoma tend to have higher costs compared with those with indolent lymphomas [27]. This cost seems to rise significantly when the patients relapse after first-line therapy. Purdum et al reported that major drivers of cost in patients requiring second-line treatment were the cost of hematopoietic stem cell transplantation and the cost of second-line chemotherapy [28].

Study strengths and limitations

Our study was based on large, pooled data, which increased its power. The NIS is generated at the hospital level and weighted to reflect the US population thus a reliable source to make hypotheses about population-wide problems in the inpatient setting making it more generalizable. One limitation is that the NIS is subject to coding errors, which can affect the quality and reliability of the data. Another limitation is that the NIS does not include information on outpatient care. We are also unable to evaluate laboratory parameters to determine the severity of hypercalcemia. There is no coding of therapeutic interventions hence the impact of specific treatments cannot be ascertained. We acknowledge that our conclusions are only postulates and proof of causality is lacking due to limitations of our methodology.

## Conclusions

In the US-based study, among patients with DLBCL admitted for inpatient chemotherapy, those with hypercalcemia compared to a cohort without, had higher odds of; all-cause mortality, TLS, AKI, acute encephalopathy, acute metabolic acidosis, hyperkalemia, and acute respiratory failure as well as increased length of stay by one day and in total charge by US$ 12,501. There was a linear positive correlation between LOS and the frequency of TLS-related complications as well as complications related to respiratory failure and at all levels of LOS, patients with hypercalcemia had higher composite complication rates compared to the cohorts without hypercalcemia. There was no difference in odds of septic shock, Clostridiodes difficile infection, and acute VTE between the two groups. These conclusions are hypothesis-generating and may need further prospective or controlled studies for confirmation. The findings, however, underscore the importance of closely monitoring and managing hypercalcemia in patients with DLBCL to improve their clinical outcomes. Future research should focus on prospective studies investigating inpatient outcomes based on the severity of hypercalcemia and the effect of specific chemotherapy regimens. Additionally, exploring potential genetic factors and the underlying reasons for the observed racial disparities in the prevalence of hypercalcemia could provide valuable insights for personalized treatment strategies.

## Appendices

**Table 3:** Key ICD-10 and PCS codes and corresponding diagnosis and procedures ICD: International Classification of Diseases, Tenth Revision; PCS:

ICD-10 code	Diagnosis/procedure
C83.3	Diffuse Large B Cell Lymphoma
Z51.1	Admission for chemotherapy
E83.52	Hypercalcemia
E87.5	Hyperkalemia
E87.2	Metabolic acidosis
G93.41	Acute encephalopathy
E88.3	Tumor lysis syndrome
N17	Acute kidney injury
I82	Venous thromboembolic events
A047	Clostridiodes dificile infection
J96, 5A1955Z, 5A1935Z, 5A1945Z	Respiratory failure
R65.21	Septic shock
E21, E34.2	Hyperparathyroidism
